# Use machine learning to help identify possible sarcopenia cases in maintenance hemodialysis patients

**DOI:** 10.1186/s12882-023-03084-7

**Published:** 2023-02-14

**Authors:** Hualong Liao, Yujie Yang, Ying Zeng, Ying Qiu, Yang Chen, Linfang Zhu, Ping Fu, Fei Yan, Yu Chen, Huaihong Yuan

**Affiliations:** 1 Department of Nephrology, West China Hospital, Sichuan University/ West China School of Nursing, Sichuan University, Chengdu, 610041 Sichuan China; 2 Department of Applied Mechanics, College of Architecture and Environment, Sichuan University, Chengdu, 610065 Sichuan China; 3 Kidney Research Laboratory, Division of Nephrology, West China Hospital of Sichuan University, Chengdu, 610041 Sichuan China; 4 Chongqing Municipality Clinical Research Center for Geriatric Diseases, Chongqing University Three Gorges Hospital, School of Medicine, Chongqing University, Chongqing, 404000 China

**Keywords:** Maintenance Hemodialysis, Sarcopenia, Machine Learning, Identification

## Abstract

**Background:**

Maintenance hemodialysis (MHD) patients often suffer from sarcopenia, which is strongly associated with their long-term mortality. The diagnosis and treatment of sarcopenia, especially possible sarcopenia for MHD patients are of great importance. This study aims to use machine learning and medical data to develop two simple sarcopenia identification assistant tools for MHD patients and focuses on sex specificity.

**Methods:**

Data were retrospectively collected from patients undergoing MHD and included patients’ basic information, body measurement results and laboratory findings. The 2019 consensus update by Asian working group for sarcopenia was used to assess whether a MHD patient had sarcopenia. Finally, 140 male (58 with possible sarcopenia or sarcopenia) and 102 female (65 with possible sarcopenia or sarcopenia) patients’ data were collected. Participants were divided into sarcopenia and control groups for each sex to develop binary classifiers. After statistical analysis and feature selection, stratified shuffle split and Synthetic Minority Oversampling Technique were conducted and voting classifiers were developed.

**Results:**

After eliminating handgrip strength, 6-m walk, and skeletal muscle index, the best three features for sarcopenia identification of male patients are age, fasting blood glucose, and parathyroid hormone. Meanwhile, age, arm without vascular access, total bilirubin, and post-dialysis creatinine are the best four features for females. After abandoning models with overfitting or bad performance, voting classifiers achieved good sarcopenia classification performance for both sexes (For males: sensitivity: 77.50% ± 11.21%, specificity: 83.13% ± 9.70%, F1 score: 77.32% ± 5.36%, the area under the receiver operating characteristic curves (AUC): 87.40% ± 4.41%. For females: sensitivity: 76.15% ± 13.95%, specificity: 71.25% ± 15.86%, F1 score: 78.04% ± 8.85%, AUC: 77.69% ± 7.92%).

**Conclusions:**

Two simple sex-specific sarcopenia identification tools for MHD patients were developed. They performed well on the case finding of sarcopenia, especially possible sarcopenia.

## Introduction

The prevalence and medical resource consumption of chronic kidney disease (CKD) are rapidly increasing with the incidence of diabetes mellitus (DM), obesity, and hypertension. Furthermore, CKD plays an important role in increasing the prevalence of cardiovascular diseases and patient mortality. The criteria for CKD is that there are markers of kidney damage or glomerular filtration rate < 60 ml/min per 1.73 m^2^ for > 3 months. Its latest classification includes cause of disease, level of GFR (6 categories), and level of albuminuria (3 categories) [1]. When CKD progresses to kidney failure (glomerular filtration rate < 15 ml/min per 1.73 m^2^), renal replacement therapies, including kidney transplantation and dialysis, should be performed. Maintenance hemodialysis (MHD) is the usual choice for dialysis. However, it is not a treatment measure. Moreover, patients undergoing MHD suffer from many comorbidities and nutritional problems, which is becoming a common challenge worldwide [2]. MHD also increases whole-body and muscle proteolysis rates and elevates the net whole-body and muscle protein loss [3], resulting in sarcopenia. Sarcopenia is defined as age-related loss of muscle mass, plus low muscle strength, and/or low physical performance [4]. Additionally, in the latest 2019 sarcopenia consensus updated by Asia working group for sarcopenia (AWGS 2019), possible sarcopenia was introduced and defined as low muscle strength or physical performance. There is also a similar term and definition in the sarcopenia consensus published by European Working Group on Sarcopenia in Older People [5]. It is proven that sarcopenia is strongly associated with long-term mortality and cardiovascular events in MHD patients [6]. Furthermore, the average prevalence of sarcopenia in dialysis patients is 28.5% [7].

Though possible sarcopenia is recommended for using in primary health care and preventive services, MHD patients are more prone to suffering from sarcopenia and the poorer physical function is worse for their survival. A longitudinal study of patients with kidney failure has shown that low muscle strength predicts mortality better than low muscle mass [8]. Therefore, the diagnosis and treatment of sarcopenia, especially possible sarcopenia for MHD patients are very important [9]. Identifying possible sarcopenia cases may help clinicians identify potential underlying causes and provide appropriate personalized interventions for patients to reverse possible sarcopenia.

Bioimpedance analysis and dual-energy x-ray absorptiometry are two recommended methods for measuring skeletal muscle mass in AWGS 2019 [4]. But the instruments are expensive and not suitable for all MHD patients to measure. Handgrip strength (HGS) and physical performance examinations are easy to perform in outpatient and community. However, some biochemical indices that have been reported to be related to sarcopenia are ignored in sarcopenia assessment. As modern clinical medical systems have recorded many health and medical data, secondary use of medical data may bring discoveries and understanding about illness and treatment, enhance healthcare experiences, and increase the efficiency of healthcare systems [10]. Additionally, MHD patients are asked to undergo centralized examinations regularly. Hence, they own many normalized medical data with potential medical values. The cohort is relatively fixed because MHD patients undergo regular dialysis, making it easier to conduct long-term observations and research.

Machine learning, one of the main methods for data mining in our daily life, has been widely applied in medical data analysis with the development of information technology and artificial intelligence [11]. It can assist in clinical tasks such as aided diagnosis, disease and prognosis prediction, and other decision-making tasks. Clinicians can take the results of machine learning as additional references and react to disease identification and development, or any possible poor prognosis accordingly [12]. Electronic medical record data, nutrition intake, physical activity performance measurement results, medical history, socio-demographic and primary care data have been used to develop machine learning risk prediction or identification models and also find the risk factors about sarcopenia [13, 14].

Similarly, the medical data of MHD patients can be used to conduct relative research, too. However, few studies have been conducted so far. In order to help clinicians know MHD patients’ sarcopenia states early and accurately and get references for clinical decision making in sarcopenia treatment, this study aims to use machine learning methods to develop simple and accurate sarcopenia assistant identification tools for MHD patients of both sexes.

## Methods

### Data collection and measurements

Data were retrospectively collected from patients undergoing MHD at Wenjiang Hemodialysis Center in the Department of Nephrology in West China Hospital, Sichuan University, Chengdu, China. All participants were over 18 years old without any mental disorder, and kept in-center maintenance hemodialysis for at least 3 months. Finally, a total of 140 male (49 with possible sarcopenia, 9 with sarcopenia or sever sarcopenia) and 102 female (53 with possible sarcopenia, 12 with sarcopenia or sever sarcopenia) MHD patients participated in this study. Sarcopenia diagnosis was carried out via AWGS 2019 [4]. The data set involved a total of 84 features, including patients’ basic information, body measurement results, and laboratory findings. Basic information was obtained through patients' medical archives, while body measurement results and laboratory findings came from the latest centralized examination at the hemodialysis center before data collection. HGS was tested by the arm without vascular access. Laboratory findings included routine blood examination, hepatic and renal function indicators, serum inorganic salts, and parathyroid hormone (PTH). Since urea and creatinine were measured again after hemodialysis, participants’ urea and creatinine after hemodialysis were also collected. All these features were carried forward as possible risk factors of sarcopenia in MHD patients.

### Data processing

Similar to Hassler’s work [15], this study combined patients with different sarcopenia states as the sarcopenia group. In comparison, patients without sarcopenia were regarded as the control group to model a binary classifier for identifying whether a new patient had sarcopenia. The cutoff values of sarcopenia diagnosis measures differ between males and females due to the physical differences between both sexes. Hence, statistical analysis and classifier models were also studied separately for special reference to sex specificity.

All text results have been converted to digital classification values for subsequent processing. Sarcopenia cases were given the label “1” while controls were “0”. The original dataset had missing values (eight features and seven samples had missing values) because a few individuals had not taken all body measurements and blood examinations for various reasons. Hence, to process missing values, this study inferred missing values from the existing data to retain potentially valuable data as much as possible. For a sample with one missing value of a feature, other samples of the same sex and sarcopenia state that matched with the sample were selected. Then the missing value imputation was conducted using the mean of the selected samples’ same feature without missing values. After the imputation, the data format was unified into the original format.

Exploratory data analysis was then conducted to examine the unadjusted associations between sarcopenia and the risk factors in different sex groups. According to the distributions of different features, unadjusted tests for differences in each feature between sarcopenia and control groups were carried out via Chi-square test, Student’s t-test, or the Mann–Whitney U-test. Statistical analysis was performed in SPSS, version 16.0 (SPSS Inc., Chicago, IL, USA). *P* < 0.05 means that there is a statistically significant difference between two groups.

### Feature selection

Sarcopenia can be assessed through several tests about muscle strength and mass and statistical analysis may find many features with *P* < 0.05. Using all these features to develop machine learning classifiers is not convenient enough to apply in clinical practice. Therefore, a simple and efficient machine learning identification model using a small number of features is preferred. Besides, the above statistical analysis only considered the relationship between a single feature and sarcopenia. Hence, the impact of combinations of different features on classifying sarcopenia is unclear. In order to develop a simple machine learning model, feature importance calculated by tree-based machine learning models, which represents features’ contribution to the classification model [16], was used for feature selection. The importance values based on Random Forest (RF) model with default hyper-parameters were calculated. Considering that feature importance values vary with tree-based machine learning models and different hyper-parameters, lasso regression, which does not depend on any machine learning models, was also used in this study. Feature importance calculation and lasso regression programs were all performed via Python 3.

### Identification models based on machine learning

**Fig. 1:**
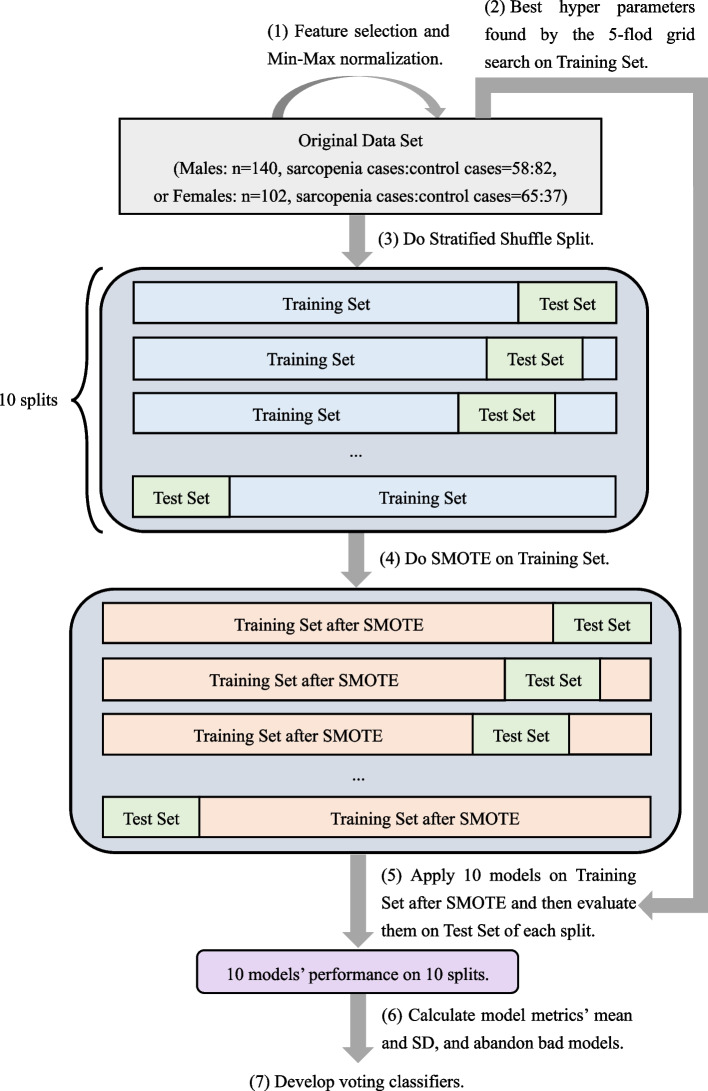
The complete process of sarcopenia classifier development

Ten commonly used binary machine learning methods were performed in this study, including K-Nearest Neighbor (KNN), Gaussian Naive Bayes (GaussianNB), Logistic Regression (LR), Support Vector Machine (SVM), Multi-layer Perceptron (MLP), and five tree-based models: Decision Tree (DT), Random Forest (RF), Adaptive Boosting (Adaboost), Gradient Boosting Decision Tree (GBDT), and Light Gradient Boosting Machine (LGBM). The complete sarcopenia classifier development process was shown in Fig. 1 and explained in detail as follows. Machine learning programs were all performed via Python 3.

Min–Max normalization was conducted to transfer feature values into a range of 0 to 1, making machine learning models converge more quickly and easily. Then, 80 percentage of the original data set of a total of 140 male samples and 102 female samples was divided into the training set and the rest was the test set, both retaining the same proportion of patients with sarcopenia as the original data set. The training set was used for doing a 5-fold grid search to find the best hyper-parameters of each machine learning method. The data size was a little small because the data did not come from a large-scale hemodialysis center. In addition, the participants were divided into two parts according to their sexes. Hence, the data size of one sex group became smaller. The results of one split and training may be less representative and unstable. Moreover, machine learning models may not gain good generation power using such a data set [17]. Therefore, stratified shuffle split method was used in model development and evaluation to reduce the influence of small data size. It is similar to cross-validation. However, the main difference is that it uses the sampling method with return and therefore one sample may be selected to compose the test set many times. The data set was split 10 times using stratified shuffle split method in this study. The data size ratio of the training set to test set was still set to four to one and the proportion of non-sarcopenia and sarcopenia cases retained the same.

Notice that the class distribution of the original data set was a little imbalanced (the prevalence of sarcopenia was 41.43%, 58/140 in males and 63.73%, 65/102 in females). Hence, a resampling method for the minority class, Synthetic Minority Oversampling Technique (SMOTE) was conducted to increase the data size and keep the data distribution balanced. It manually generates minority class samples by interpolating between several neighboring minority class examples instead of simply creating copies [18], which lets machine learning models pay balanced attention to sarcopenia and control groups and can avoid over-generalization in some degree. SMOTE did not change the true prevalence in the original data set significantly because the proportion of these two groups was not too imbalanced. Moreover, SMOTE was only done on the training set but not the original data set because using real-world data in test set to reflect the performance of machine learning classifiers in clinical practice is more preferred. After SMOTE, ten machine learning algorithms used their best hyper-parameters tuned by the 5-fold grid search to develop sarcopenia classifiers.

The true disease states of the patients in test set were used to obtain the true-negative rates (TNR, also called specificity), precision scores (the proportion of people classified as sarcopenia cases to those who are really assessed as sarcopenia cases) and true-positive rates (TPR, also called recall score and sensitivity) of ten models. Sensitivity, specificity, F1 score (2 * precision * sensitivity / (precision + *sensitivity)*) and the area under the receiver operating characteristic curve (AUC), were used together to evaluate classifiers’ performance. Other evaluation metrics for classification included the accuracy of training set and test set and the absolute of accuracy difference between these two sets. The absolute of accuracy difference was used to judge whether a model occurs overfitting. Machine learning models achieve their best performance when all metrics’ values are 1, except the absolute of accuracy difference is expected to be as close to 0 as possible [19]. If the absolute value of accuracy difference is too large, it indicates that this model occurs overfitting or has poor generalization power, and this model should be abandoned. Others with poor classification evaluation metrics will also be excluded.

After each split and modeling, every machine learning model’s performance was evaluated via the metrics mentioned above. For one metric, each model had 10 values. Therefore, the mean and SD values of these metrics were calculated. Notice that each model has different performance with various feature sets and models may not perform very well with such a data size, which makes it inconvenient to compare the classification performance of kinds of feature sets. Hence, in order to combine kinds of machine learning classifier methods, improve the final performance, and find the best feature set, the voting classifiers were proposed. The voting classifiers calculated the average probabilities that each model judged every sample as sarcopenia case in test set. If a mean value was larger than or equal to 0.5, the voting classifiers would consider the sample as a sarcopenia case. The voting classifiers combined several machine learning classifiers’ results, and also had the same evaluation metrics.

## Results

Table 1 reported descriptive statistics on the male and female MHD patients’ basic information, body measurement results and laboratory findings, stratified by sarcopenia cases versus controls. Continuous and summary statistics were expressed as mean ± standard deviation (SD) for normally distributed features and as median with interquartile range (IQR) when the normality assumption was violated. Classification statistics were expressed as n (%). Unadjusted statistical analysis found 19 features with statistically significant differences between two male groups and 13 in two female groups.

**Table 1:** Statistical analysis results of data features between sarcopenia and control groups of different sexes

Features	Male			Female		
	Control Group (*n* = 82)	Sarcopenia Group (*n* = 58)	*P*	Control Group (*n* = 37)	Sarcopenia Group(*n* = 65)	*P*
Basic information						
Age, years	45.90 ± 11.04	61.00 ± 12.23	<0 .001^***^	48.57 ± 10.18	57.28 ± 14.23	0.001^**^
Diabetes mellitus (DM)			0.006^**^			0.327
No	68(82.90%)	36(62.07%)		31(83.80%)	48(73.80%)	
Yes	14(17.10%)	22(37.93%)		6(16.20%)	17(26.20%)	
Hypertension			0.833			0.464
No	13(22.41%)	16(19.50%)		10(27.00%)	13(20.00%)	
Yes	45(77.59%)	66(80.50%)		27(73.00%)	52(80.00%)	
Vascular access type			0.022^*^			0.453
Arteriovenous fistula (AVF)	78(95.10%)	48(82.80%)		31(83.80%)	49(75.40%)	
Central venous catheter (CVC)	4(4.90%)	10(17.20%)		6(16.20%)	16(24.60%)	
Dialysis duration, months	36.00(47.00)	36.00(50.00)	0.358	43.00(39.50)	55.00(51.00)	0.165
Body measurement results						
Strength, assistance with walking, rise from a chair, climb stairs and falls (SARC-F),	10.00(10.00)	10.00(10.00)	0.013^*^	10.00(0.00)	10.00(1.00)	0.014^*^
Calf circumference (CC), cm	34.00(4.00)	32.50(5.00)	0.062	30.00(4.50)	30.00(4.75)	0.392
Waist circumference (WC), cm	88.50(12.50)	90.00(12.00)	0.612	81.80 ± 9.37	78.57 ± 9.39	0.098
Hip circumference (HC), cm	95.00(8.50)	95.00(11.00)	0.806	89.50(9.00)	86.50(8.00)	0.154
Arm without vascular access (AWVA)			1			< 0.001^***^
The right arm	48(58.50%)	34(58.60%)		14(37.80%)	49(75.40%)	
The left arm	34(41.50%)	24(41.40%)		23(62.20%)	16(24.60%)	
Handgrip strength (HGS), kg	36.36 ± 6.80	25.98 ± 6.74	<0 .001^***^	24.43 ± 4.69	16.60 ± 5.18	< 0.001^***^
6-m walk, m/s	1.10(0.13)	0.93(0.18)	<0 .001^***^	1.05(0.11)	0.89(0.19)	< 0.001^***^
Skeletal muscle index (SMI), kg/m^2^	8.54 ± 0.82	8.13 ± 0.94	0.007^**^	6.88 ± 0.70	6.40 ± 0.84	0.004^**^
Height, cm	169.00(8.00)	167.00(9.00)	0.017^*^	156.24 ± 5.81	153.74 ± 5.46	0.032^*^
Weight, kg	66.80(14.00)	65.30(16.30)	0.284	52.20(11.45)	51.00(10.25)	0.107
Body mass index (BMI), m/kg^2^	23.88(4.55)	23.94(3.90)	0.844	22.23 ± 3.04	21.52 ± 3.25	0.277
Laboratory findings						
Red blood cell count (RBC), 10^12^/L	3.75(0.74)	3.77(0.80)	0.936	3.51 ± 0.46	3.61 ± 0.57	0.403
Hemoglobin (HGB), g/L	113.00(19.00)	111.00(20.00)	0.783	107.51 ± 13.10	106.65 ± 14.44	0.764
Hematocrit (HCT), L/L	0.35(0.06)	0.36(0.07)	0.508	0.34 ± 0.04	0.34 ± 0.05	0.786
Mean corpuscular volume (MCV), fL	93.30(6.90)	93.80(6.10)	0.241	96.20(6.05)	94.20(6.65)	0.078
Mean corpuscular hemoglobin (MCH), pg	30.10(2.40)	30.20(2.80)	0.245	30.20(2.00)	30.30(2.30)	0.209
Mean corpuscular hemoglobin concentration (MCHC), g/L	322.00(20.00)	322.00(18.00)	0.911	317.49 ± 9.79	317.77 ± 11.41	0.9
Red blood cell distribution width—coefficient of variation (RDW-CV), %	14.90(2.10)	14.90(2.40)	0.21	14.90(1.10)	15.30(2.15)	0.048^*^
Red blood cell distribution width—standard deviation (RDW-SD), fL	48.30(8.60)	49.20(8.50)	0.581	51.40(5.15)	50.70(6.05)	0.223
Platelet count (PLT), 10^9^/L	182.00(87.00)	161.00(86.00)	0.23	171.00(104.00)	162.00(82.50)	0.371
White blood cell count (WBC), 10^9^/L	6.29(2.13)	6.38(2.42)	0.869	6.31(2.71)	5.99(2.42)	0.197
Neutrophil percentage (NEUT%), %	67.26 ± 7.46	68.67 ± 7.46	0.27	67.70 ± 5.76	68.20 ± 9.60	0.744
Lymphocyte percentage (LY%), %	23.10 ± 6.22	21.02 ± 6.83	0.063	23.28 ± 5.23	22.54 ± 7.89	0.569
Monocyte percentage (MO%), %	5.30(2.20)	5.70(2.30)	0.156	4.80(2.30)	5.60(2.25)	0.215
Eosimophil percentage (EOS%), %	3.00(3.00)	2.90(3.60)	0.808	2.20(1.90)	2.60(3.05)	0.978
Basophil percentage (BASO%), %	0.30(0.30)	0.30(0.30)	0.395	0.30(0.20)	0.20(0.25)	0.164
Absolute neutrophil count (ANC), 10^9^/L	4.29(1.92)	4.22(1.80)	0.762	4.14(1.98)	3.75(1.84)	0.16
Absolute lymphocyte count (ALC), 10^9^/L	1.41(0.73)	1.28(0.60)	0.195	1.37(0.60)	1.23(0.89)	0.132
Absolute monocytes count (AMC), 10^9^/L	0.32(0.20)	0.38(0.15)	0.121	0.29(0.19)	0.31(0.17)	0.859
Absolute eosinophils count (AEC), 10^9^/L	0.18(0.19)	0.18(0.21)	0.601	0.13(0.12)	0.14(0.18)	0.805
Absolute basophil count (ABC), 10^9^/L	0.02(0.02)	0.02(0.02)	0.311	0.02(0.02)	0.01(0.01)	0.181
Total bilirubin (TBIL), μmol/L	6.70(3.90)	7.90(3.90)	0.1	7.30(2.95)	6.20(3.40)	0.048^*^
Direct bilirubin (DBIL), μmol/L	2.00(1.30)	2.60(1.30)	0.018^*^	1.90(1.20)	1.70(1.10)	0.473
Indirect bilitubin (IBIL), μmol/L	4.70(2.70)	5.50(2.60)	0.208	5.10(2.35)	4.40(2.60)	0.024^*^
Alanine amiotransferase (ALT), IU/L	12.00(9.00)	14.00(10.00)	0.037^*^	11.00(8.00)	11.00(7.00)	0.43
Aspartate aminotransferase (AST), IU/L	12.00(7.00)	15.00(8.00)	0.002^**^	17.00(8.00)	16.00(4.50)	0.837
Aspartate aminotransferase/alanine amiotransferase (AST/ALT)	1.15(0.55)	1.07(0.49)	0.901	1.30(0.64)	1.40(0.58)	0.125
Total protein (TP), g/L	71.08 ± 4.71	70.38 ± 3.65	0.33	71.20(7.35)	70.50(7.15)	0.23
Albumin (ALB), g/L	43.80(4.90)	43.60(4.00)	0.258	44.00(4.40)	42.50(5.90)	0.021^*^
Globulin (GLB), g/L	26.77 ± 3.68	26.71 ± 3.55	0.922	28.13 ± 4.78	28.18 ± 5.04	0.956
Albumin/globulin (A/G)	1.69 ± 0.27	1.67 ± 0.28	0.702	1.53(0.42)	1.50(0.44)	0.337
Fasting blood glucose (FBG), mmol/L	5.12(0.93)	5.61(2.76)	0.007^**^	4.91(0.96)	5.09(1.57)	0.254
Pre-dialysis urea (pre-URE), mmol/L	24.07 ± 5.84	22.65 ± 6.46	0.178	22.57 ± 5.08	21.65 ± 6.42	0.429
Pre-dialysis creatinine (pre-CRE), μmol/L	1140.94 ± 234.52	947.41 ± 237.84	<0 .001^***^	924.11 ± 174.82	818.63 ± 203.91	0.01^*^
Cystatin C (CysC), mg/L	6.93(1.33)	6.82(1.86)	0.454	6.40 ± 1.04	6.72 ± 1.06	0.142
Uric acid (UA), μmol/L	453.13 ± 97.99	432.09 ± 103.48	0.223	419.65 ± 79.52	388.97 ± 108.40	0.105
Triglyceride (TG), mmol/L	1.85(1.23)	1.45(1.13)	0.035^*^	1.39(1.56)	1.35(0.75)	0.459
Cholesterol (CHOL), mmol/L	3.65(1.15)	3.47(0.92)	0.198	4.01(1.19)	3.77(1.14)	0.524
High density lipoprotein cholesterol (HDL-C), mmol/L	0.84(0.33)	0.95(0.45)	0.014^*^	1.09 ± 0.30	1.19 ± 0.35	0.118
Low density lipoprotein (LDL), mmol/L	1.96(0.89)	1.85(0.87)	0.341	2.11(0.93)	1.94(0.96)	0.648
Alkaline phosphatase (ALP), IU/L	75.00(32.00)	77.00(32.00)	0.249	84.00(53.50)	84.00(51.00)	0.981
γ-glutamyl transpeptadase (GGT), IU/L	18.00(16.00)	19.00(22.00)	0.4	13.00(11.00)	13.00(15.00)	0.425
Serum sodium (s-Na), mmol/L	138.69 ± 2.23	138.50 ± 2.64	0.644	138.13 ± 2.19	137.27 ± 3.26	0.116
Serum potassium (s-K), mmol/L	4.68(0.80)	4.82(1.18)	0.842	4.98 ± 0.68	4.80 ± 0.74	0.231
Serum chloride (s-Cl), mmol/L	97.78 ± 3.26	97.74 ± 4.38	0.946	98.63 ± 4.00	98.17 ± 4.13	0.586
Carbon dioxide combining power (CO_2_CP), mmol/L	21.55 ± 2.66	22.25 ± 2.72	0.131	21.57 ± 3.26	21.59 ± 3.15	0.975
Anion gap (AG), mmol/L	24.11 ± 3.05	23.27 ± 4.12	0.192	22.91 ± 3.22	22.32 ± 4.09	0.452
Serum β-hydroxybutyrate (β-HBA), mmol/L	0.10(0.07)	0.09(0.10)	0.882	0.11(0.09)	0.11(0.10)	0.958
Serum calcium (s-Ca), mmol/L	2.29 ± 0.20	2.27 ± 0.21	0.69	2.31 ± 0.16	2.31 ± 0.20	0.863
Serum magnesium (s-Mg), mmol/L	1.05 ± 0.12	1.06 ± 0.13	0.478	1.07(0.21)	1.03(0.22)	0.235
Serum inorganic phosphorus (s-IP), mmol/L	1.98 ± 0.48	1.76 ± 0.50	0.01^*^	1.87 ± 0.47	1.82 ± 0.49	0.576
Serum iron (s-I), μmol/L	14.10(8.70)	14.10(6.20)	0.966	14.00(7.30)	11.80(7.45)	0.34
Total iron binding capacity (TIBC), μmol/L	46.20(13.40)	45.20(12.00)	0.509	41.90(13.35)	42.67(13.40)	0.694
Transferrin saturation (TSAT), %	30.48(17.60)	32.40(12.40)	0.933	32.40(19.50)	27.10(14.05)	0.233
Parathyroid hormone (PTH), pmol/L	36.56(40.89)	24.87(23.80)	0.001^**^	37.06(35.02)	36.70(36.18)	0.71
Serum ferritin (s-Fe), μg/L	249.50(373.80)	331.50(378.80)	0.214	450.30(516.00)	300.00(351.15)	0.244
C-reactive protein (CRP), μg/L	4.28(5.64)	4.16(9.14)	0.988	2.15(4.54)	2.84(5.37)	0.878
β2-microglobulin (β2-MG), mg/mL	40.56 ± 10.54	42.04 ± 13.76	0.472	41.50(17.25)	39.60(15.20)	0.802
Post-dialysis urea (post-URE), mmol/L	7.90(2.90)	7.90(4.40)	0.535	5.40(2.15)	4.90(2.00)	0.132
Post-dialysis creatinine (post-CRE), μmol/L	450.12 ± 132.64	371.53 ± 105.75	<0 .001^***^	276.00(72.50)	248.00(90.00)	0.009^**^

^*^P values less than 0.05

^*^^*^P values less than 0.01

^*^^**^P values less than 0.001

After calculating the feature importance values and absolute weights of lasso regression of 84 features, the average ranking was gotten. The ranking results of features in two sex groups shown in Table 2 were in descending order of the average ranking. Only top 15 ranking results were given.

**Table 2:** Top 15 features ranked by the average ranking of feature importance and absolute feature weight of lasso regression

Male							Female						
Feature	IRF^a^	RIRF^b^	AFWL^c^	RAFWL^d^	AR^e^	*P*	Feature	IRF	RIRF	AFWL	RAFWL	AR	*P*
6-m walk	0.1987	1	1.1335	1	1	< 0.001	HGS	0.1440	1	1.1379	1	1	< 0.001
HGS	0.1470	2	1.0320	2	2	< 0.001	6-m walk	0.1233	2	0.6048	2	2	< 0.001
age	0.0600	3	0.5118	3	3	< 0.001	AWVA	0.0241	3	0.4042	3	3	< 0.001
FBG	0.0236	4	0	9	6.5	0.007	TBIL	0.0229	4	0	9	6.5	0.048
PTH	0.0220	5	0	9	7	0.001	TP	0.0186	5	0	9	7	0.23
pre-CRE	0.0217	6	0	9	7.5	< 0.001	TG	0.0177	6	0	9	7.5	0.459
AG	0.0197	7	0	9	8	0.192	AST/ALT	0.0169	7	0	9	8	0.125
post-CRE	0.0188	8	0	9	8.5	< 0.001	SMI	0.0154	8	0	9	8.5	0.004
AST	0.0137	10	0	9	9.5	0.002	CysC	0.0144	9	0	9	9	0.142
LY%	0.0125	11	0	9	10	0.063	WC	0.0140	10	0	9	9.5	0.098
TP	0.0115	12	0	9	10.5	0.33	post-CRE	0.0134	11	0	9	10	0.009
RDW-CV	0.0110	13	0	9	11	0.21	age	0.0132	12	0	9	10.5	0.001
CysC	0.0107	14	0	9	11.5	0.454	s-Fe	0.0129	13	0	9	11	0.244
height	0.0105	15	0	9	12	0.017	weight	0.0127	14	0	9	11.5	0.107
s-Mg	0.0103	16	0	9	12.5	0.478	pre-CRE	0.0126	15	0	9	12	0.01

*HGS* Handgrip strength, *FBG* Fasting blood glucose, *PTH* Parathyroid hormone, *pre-CRE* Pre-dialysis creatinine, *AG* Anion gap, *post-CRE* Post-dialysis creatinine, *AST* Aspartate aminotransferase, *LY%* Lymphocyte percentage, *TP* Total protein, *RDW-CV* Red blood cell distribution width—coefficient of variation, *CysC* Cystatin C, *s-Mg* Serum magnesium, *AWVA* Arm without vascular access, *WC* Waist circumference, *TBIL* Total bilirubin, *TG* Triglyceride, *AST/ALT* Aspartate aminotransferase/alanine aminotransferase, *SMI* Skeletal muscle index, *s-Fe* Serum ferritin

^a^Importance value calculated by RF

^b^Ranking of importance value calculated by RF

^c^Absolute feature weight of lasso regression

^d^Ranking of absolute feature weight of lasso regression

^e^Average ranking

The 6-m walk and HGS were considered to be the most two important features for identifying possible sarcopenia and sarcopenia after feature selection, and *P* values also showed statistically significant differences of these two features in both sexes. According to AWGS 2019, possible sarcopenia can be assessed by any one of these two items’ test results, confirming the reliability of the feature importance and weight ranking method in degrees.

Among the top 15 features sorted by average ranking, some features, like age, pre-dialysis creatinine (pre-CRE), and post-dialysis creatinine (post-CRE), had significant differences. Meanwhile, other features with *P* > 0.05, such as anion gap (AG), total protein (TP), and triglyceride (TG), might also be helpful to the development of the machine learning classifier because of their high average rankings. Therefore, to obtain a simple and efficient machine learning identification model different from existing means of diagnosis, top 3, 4, 5 feature and top 3, 4, 5 feature with statistically significant differences (*P* < 0.05) after excluding 6-m walk, HGS, and skeletal muscle index (SMI) in Table 2, were selected for modeling. Their performance was compared to find the best feature set for sarcopenia identification.

**Fig. 2:**
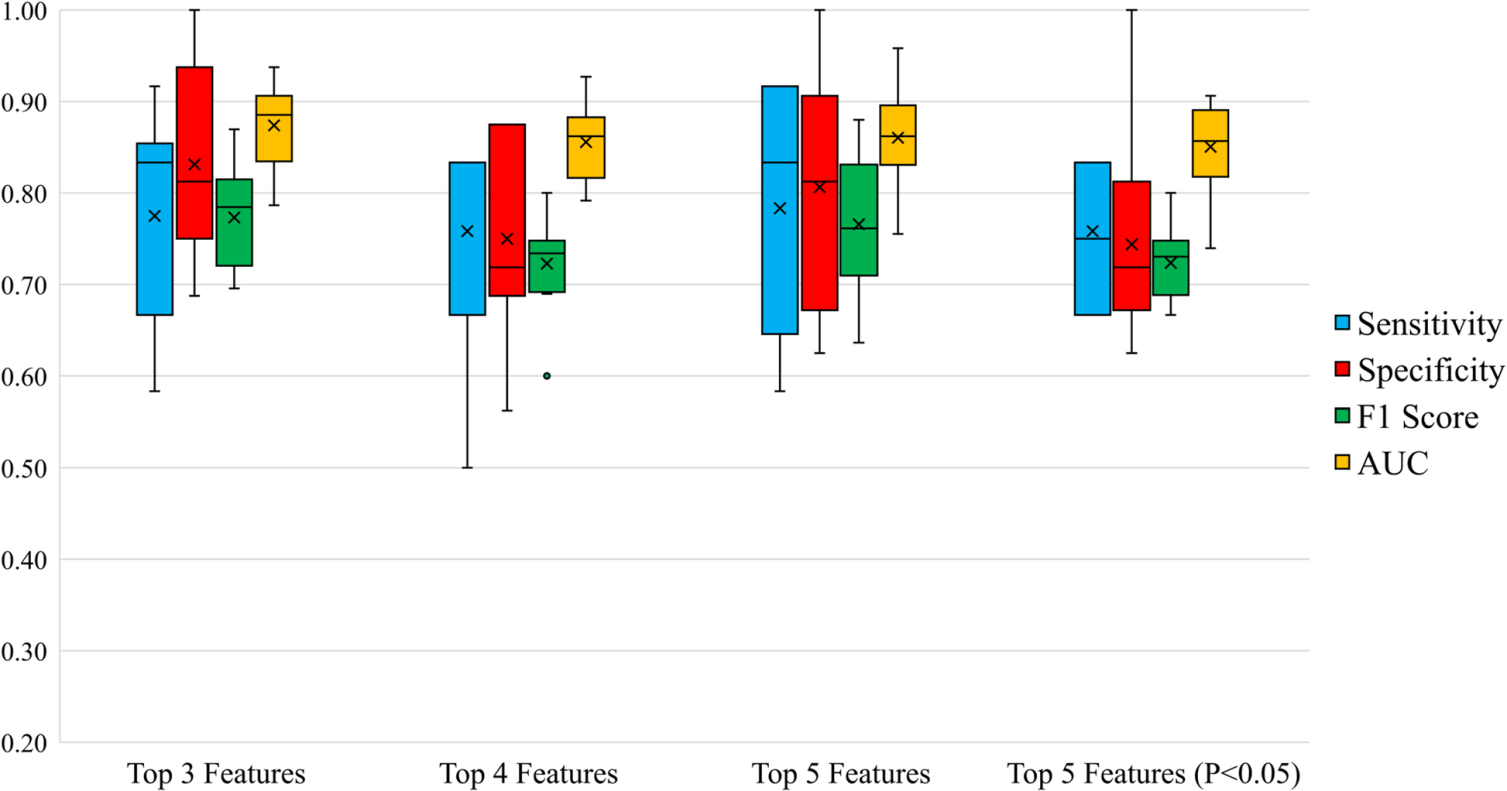
The box plot of voting classifier’s evaluation metrics about six feature sets in males.  × : the mean value mark. ○: the outliers

**Fig. 3:**
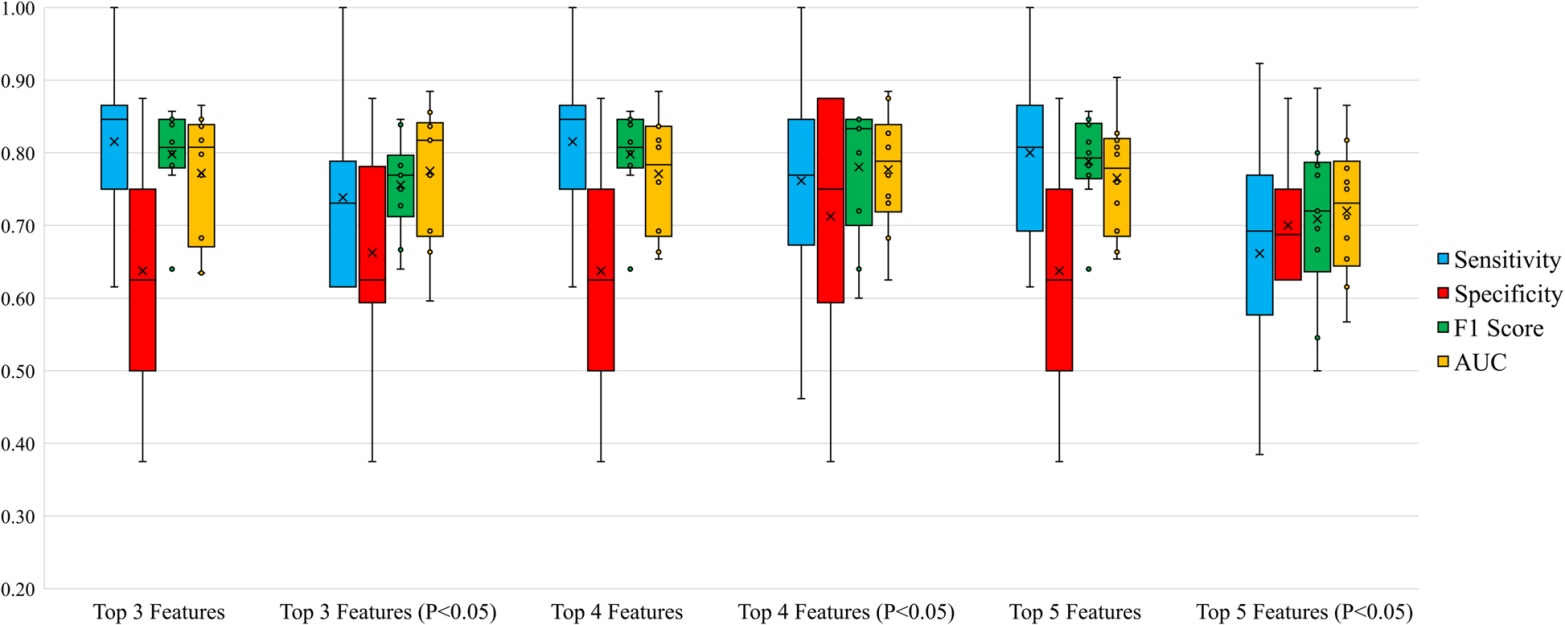
The box plot of voting classifier’s evaluation metrics about six feature sets in females.  × : the mean value mark. ○: the outliers

After abandoning models occurring overfitting and unacceptable bad performance, all evaluation metrics of voting classifiers using top 3, 4, 5 feature sets and top 3, 4, 5 feature sets with *P* < 0.05 about 10 splits were shown in Table 3 and Table 4. The sensitivity, specificity, F1 score and AUC of voting classifiers were draw into a box plot as shown in Fig. 2 and Fig. 3. Notice that the top 4 features of male patients in Table 2 all had statistically significant differences. Hence, only 4 kinds of feature sets were compared in male MHD patients.

**Table 3:** The voting classifier’s evaluation metrics about four feature sets in male MHD patients

Metric	Top 3 Features	Top 4 Features	Top 5 Features	Top 5 Features (*P* < 0.05)
ACCTRS^a^	86.59% ± 1.89%	73.03% ± 1.41%	78.48% ± 2.82%	74.92% ± 2.41%
ACCTES^b^	80.71% ± 4.29%	75.36% ± 4.06%	79.64% ± 6.79%	75.00% ± 4.79%
AVAD^c^	6.61% ± 3.63%	4.88% ± 2.70%	8.00% ± 3.02%	3.69% ± 3.45%
Precision	79.28% ± 9.86%	70.65% ± 6.79%	77.90% ± 13.17%	70.61% ± 10.71%
Sensitivity	77.50% ± 11.21%	75.83% ± 10.83%	78.33% ± 13.54%	75.83% ± 6.92%
Specificity	83.12% ± 9.70%	75.00% ± 10.08%	80.62% ± 12.95%	74.38% ± 10.63%
F1 Score	77.32% ± 5.36%	72.28% ± 5.12%	76.58% ± 7.61%	72.36% ± 4.11%
AUC	87.40% ± 4.41%	85.57% ± 3.86%	86.04% ± 5.35%	85.05% ± 4.76%

^a^Accuracy of Training Set

^b^Accuracy of Test Set

^c^Absolute Value of Accuracy Difference between Training Set and Test Set

**Table 4:** The voting classifier’s evaluation metrics about six feature sets in female MHD patients

Metric	Top 3 Features	Top 3 Features (*P* < 0.05)	Top 4 Features	Top 4 Features (*P* < 0.05)	Top 5 Features	Top 5 Features (*P* < 0.05)
ACCTRS^a^	63.27% ± 3.54%	65.67% ± 3.57%	65.00% ± 2.69%	66.73% ± 4.28%	63.94% ± 2.24%	73.37% ± 3.25%
ACCTES^b^	74.76% ± 6.75%	70.95% ± 6.55%	74.76% ± 6.75%	74.29% ± 8.57%	73.81% ± 6.48%	67.62% ± 9.94%
AVAD^c^	14.10% ± 5.34%	8.20% ± 5.45%	12.37% ± 4.26%	12.72% ± 5.77%	11.32% ± 3.96%	11.25% ± 8.28%
Precision	79.03% ± 6.62%	78.77% ± 7.13%	79.03% ± 6.62%	81.86% ± 7.58%	78.75% ± 6.44%	77.68% ± 6.73%
Sensitivity	81.54% ± 10.43%	73.85% ± 11.51%	81.54% ± 10.43%	76.15% ± 13.95%	80.00% ± 10.99%	66.15% ± 14.68%
Specificity	63.75% ± 14.20%	66.25% ± 14.84%	63.75% ± 14.20%	71.25% ± 15.86%	63.75% ± 14.20%	70.00% ± 8.29%
F1 Score	79.81% ± 6.02%	75.59% ± 6.20%	79.81% ± 6.02%	78.04% ± 8.85%	78.84% ± 5.94%	70.89% ± 11.06%
AUC	77.21% ± 8.43%	77.50% ± 8.89%	77.12% ± 7.53%	77.69% ± 7.92%	76.54% ± 7.61%	72.02% ± 8.75%

^a^Accuracy of Training Set

^b^Accuracy of Test Set

^c^Absolute Value of Accuracy Difference between Training Set and Test Set

In Fig. 2, the top 4 feature set and top 5 feature set (*P* < 0.05) both had low F1 scores. Thus, these two sets were not recommended for sarcopenia identification. The top 3 feature set seemed to have a similar performance to the top 5 feature set. But in Table 3, the top 3 feature set achieved a little higher F1 score and AUC with lower SD than the top 5 feature set. Besides, using less features is more popular because it is easier to measure them and explain how the classifier works. Therefore, top 3 feature set became the best feature set for classifying sarcopenia in male MHD patients.

When males only used 3 features to identify sarcopenia accurately, females needed more. In Fig. 3, none of the voting classifier performed as well as the voting classifier using top 3 features in males. Only top 4 and top 5 feature sets (*P* < 0.05) got an average specificity over 70%. Compared with top 5 feature set (*P* < 0.05), the top 4 feature set (*P* < 0.05) had an advantage in all metrics except the absolute of accuracy difference. Though it had a larger IQR of specificity, it achieved better results of several classification evaluation metrics at once. Overall, the top 4 feature set (*P* < 0.05) was considered to be better than other feature sets.

The mean and SD of metrics about machine learning classifiers involved in voting classifier using the best feature set were calculated as shown in Table 5. The average sensitivity of LR was larger than Adaboost and LGBM as it identified more males with sarcopenia, while its precision, specificity and F1 score were the lowest. The performance of Adaboost was similar to LGBM’s. However, LGBM had the largest accuracy difference, which means it was not very robust. After combining these models, the voting classifier got improvement on them and kept a low absolute of accuracy difference. Its F1 score and AUC all exceeded other three models and it reduced their SD simultaneously. Besides, the voting classifier also kept high specificity as these models. The difference between voting classifier’s average specificity and sensitivity was less than 6%. Therefore, it was considered to classify sarcopenia and non-sarcopenia male patients in a balanced way. Overall, the voting classifier showed better classification performance than any single model.

**Table 5:** The performance of the voting classifier and various models that voting classifier combines

Metric	Male				Female	
	LR	Adaboost	LGBM	VC^d^	SVM	VC^d^
ACCTRS^a^	73.18% ± 3.07%	80.61% ± 3.68%	89.39% ± 1.92%	86.59% ± 1.89%	66.73% ± 4.28%	66.73% ± 4.28%
ACCTES^b^	75.36% ± 3.37%	77.86% ± 6.74%	78.93% ± 6.48%	80.71% ± 4.29%	74.29% ± 8.57%	74.29% ± 8.57%
AVAD^c^	4.77% ± 3.87%	7.55% ± 5.54%	10.47% ± 6.76%	6.61% ± 3.63%	12.72% ± 5.77%	12.72% ± 5.77%
Precision	71.28% ± 10.45%	75.37% ± 12.04%	75.39% ± 8.86%	79.28% ± 9.86%	81.86% ± 7.58%	81.86% ± 7.58%
Sensitivity	77.50% ± 14.93%	76.67% ± 12.80%	76.67% ± 13.84%	77.50% ± 11.21%	76.15% ± 13.95%	76.15% ± 13.95%
Specificity	73.75% ± 11.11%	78.75% ± 13.17%	80.62% ± 8.59%	83.12% ± 9.70%	71.25% ± 15.86%	71.25% ± 15.86%
F1 Score	72.29% ± 6.08%	74.67% ± 7.84%	75.25% ± 9.37%	77.32% ± 5.36%	78.04% ± 8.85%	78.04% ± 8.85%
AUC	86.20% ± 4.89%	85.31% ± 6.38%	85.03% ± 5.82%	87.40% ± 4.41%	77.69% ± 7.92%	77.69% ± 7.92%

^a^Accuracy of Training Set

^b^Accuracy of Test Set

^c^Absolute Value of Accuracy Difference between Training Set and Test Set

^d^Voting Classifier

In female group, SVM got the best classification performance. However, the voting classifier’s performance would become worse if SVM was combined with other models. Therefore, the voting classifier only used SVM to identify sarcopenia in female MHD patients. Though its results were not as good as the classifier for males, its F1 score of 78.04% also suggested that it could provide assistance in identification.

**Fig. 4:**
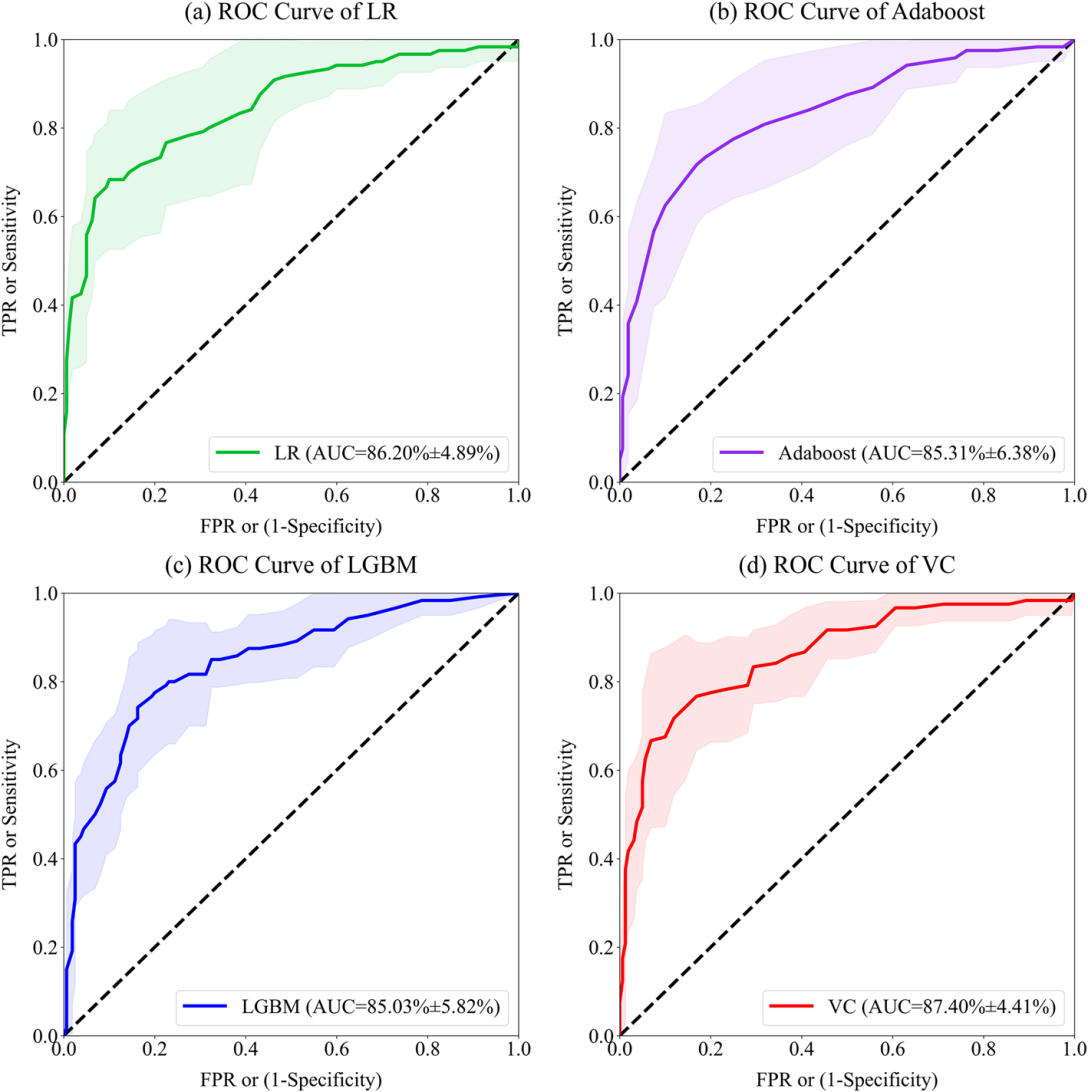
The “average” ROC curves of four classifiers using the best feature set about male patients. The solid line represents the mean value of sensitivity, and the light area can be regarded as SD values

**Fig. 5:**
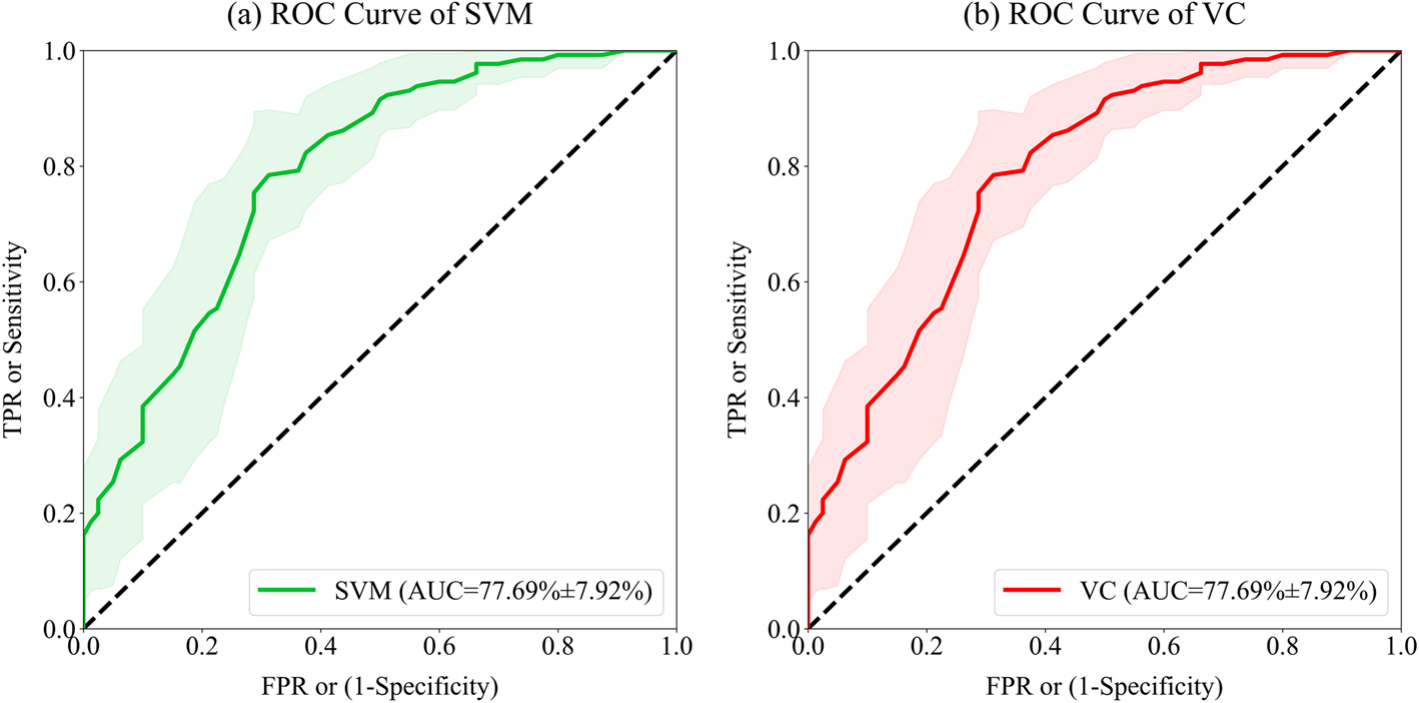
The “average” ROC curves of two classifiers using the best feature set about female patients. The solid line represents the mean value of sensitivity, and the light area can be regarded as SD values

The ROC curves of each classifier listed in Table 5 were shown in Fig. 4 and Fig. 5. Considering that a ROC curve can be plotted after each split, it is hard to evaluate several classifiers’ performance through 10 ROC curves intuitively. Therefore, an approximate ROC curve to represent 10 splits’ “average” performance of one classifier was provided. According to the principle of ROC curve, the threshold set was redesigned to obtain the FPR and TPR sequences which were used for drawing the “average” ROC curve. The mean values of FPR sequences of 10 splits were calculated to get the value series of *x* axis of the ROC curve, while the mean and SD values of TPR sequence were calculated as *y* values. Finally, according to Fig. 4, the ROC curve of voting classifier about male MHD patients was more convex to the upper left, which means a larger AUC value. Meanwhile, it retained a small TPR dispersion in degrees, that is, a low SD of sensitivity.

## Discussion

In this study, two binary classification models for the identification of sarcopenia, especially possible sarcopenia in male and female MHD patients were developed separately using features collected from the medical system. Sex-specific classifiers and features in two classifiers were mainly discussed as follows.

### Sex-specific classifiers

Previous studies on sarcopenia prediction or identification models usually do not consider sex as a very important grouping indicator, but as a risk factor. Therefore, males and females used the same risk factors in one model, and the classification performance may be not satisfactory [20]. However, studies have shown that sex-specific aging patterns involve muscle mass and quality changes [21]. Though the diagnostic methods are the same in AWGS 2019, the diagnostic cutoff values vary with sex. It is also the same in the consensus published by European Working Group on Sarcopenia in Older People [5]. Inspired by Kang’s work [13], this study focused on sex specificity and developed two classifiers. They may raise greater attention to sarcopenia among MHD patients and promote the implementation of simply sex-specific diagnosis and identification methods for sarcopenia in clinic. The hyperparameter values found through grid search and machine learning model performance were not the same for different sexes and feature sets. Therefore, the voting classifier used different feature sets and models for two sexes, reflecting the physiological differences in the onset and diagnosis of sarcopenia between males and females. However, if there is a sex-insensitive model which can identify sarcopenia in MHD patients very precisely, it will be more convenient and popular in clinical practice and is an aim of further research.

### Features in two classifiers

The features in two classifiers were selected by feature selection. Feature selection plays an important role in compressing the data processing scale. Medical data contains a large variety of examination results, medical history, nutrition intake, psychological states and so on, which are often high-dimensional. As not all data features are strongly related to the onset of sarcopenia, some unnecessary and irrelevant features need to be removed before model development. Feature selection can pre-process machine learning algorithms. Moreover, good feature selection results can improve models’ accuracy, reduce learning time, and also simplify learning results [22]. Well selected features help clinicians understand the possible underlying mechanism of sarcopenia and carry out corresponding intervention treatments. Since AWGS 2019 has recommended the sarcopenia diagnosis methods and cutoff values, it is meaningless to use the items proposed in the consensus and let machine learning to learn the existing classification rules. Therefore, HGS, 6-m walk, and SMI were excluded before developing machine learning models in this study. The features used in each classifier after feature selection were discussed as follows.

Age is a common key feature for identification in two sex groups. Sarcopenia itself is defined as an age-related geriatric syndrome [4]. It refers to the gradual decline in muscle mass, strength, and quality noted with advancing age [23]. The sarcopenia incidence increases with age [24].

In addition to age, FBG and PTH are also the most two suitable features for classification in males. Skeletal muscle is the largest insulin-sensitive tissue in the body. Hence, low muscle mass seems to result in a reduced capacity for glucose metabolism ability [25], and patients with sarcopenia have higher FBG levels [26, 27]. Other studies have shown that people with type 2 diabetes have a higher risk of developing sarcopenia [28, 29], and higher FBG levels are also a symptom of DM. Therefore, the hidden relationship between DM and sarcopenia may be explained by FBG levels. Meanwhile, according to Table 1, male patients with sarcopenia had lower PTH levels. As basal PTH levels can be modified by s-IP [30], MHD patients are prone to be at high s-IP levels [31], resulting in increased PTH secretion and possible secondary hyperparathyroidism [32]. However, male patients with sarcopenia had lower s-IP levels, which may be attributed to too little protein intake. The content of phosphorus in food is proportional to proteins, and the intake of certain proteins can help treat sarcopenia [33]. This result is consistent with the result of another study on sarcopenia in MHD patients [34].

In female group, arm without vascular access (AWVA), total bilirubin (TBIL), and post-CRE are the most three suitable features for classification besides age. AWVA between control and sarcopenia group is of statistically significant difference and has a high ranking in Table 2. Studies have shown that the HGS of AWVA is larger than the arm with vascular access [35, 36]. Although the vascular access is usually built in the non-dominant arm, varying with the vascular condition of the upper limbs and other factors, the vascular access may be built in the dominant arm. The dominant arm of each patient has not been investigated in this study. Hence, the relationship between sarcopenia and the trend of using the left arm to test HGS in female patients is unclear. Limited by this single-center study, AWVA may not be applicable for other different populations. Meanwhile, bilirubin is a potent endogenous antioxidant with anti-inflammatory, immunomodulatory and so on [37]. Low TBIL levels may lead to chronic-inflammation, and the phenomenon of chronic-inflammation or inflamm-aging is a contributor to sarcopenia [38]. The female group with sarcopenia of this study had lower TBIL levels, which is consistent with previously proven knowledge.

Female patients with sarcopenia had lower post-CRE levels in Table 1 and other studies have also shown similar results [34, 39]. Creatinine comes from both the metabolism of creatine in muscles and the meat intake. Hence, low serum creatinine levels may be regarded as a proxy for low muscle mass and protein-energy wasting, which is associated with adverse outcomes in MHD patients [40]. In both males and females, the pre-CRE and post-CRE levels of sarcopenia groups were significantly lower than those of the control groups. However, only the post-CRE level in females was selected after feature selection.

Besides, other research has shown that the modified creatinine index (mCI) and sarcopenia index (serum creatinine / CysC × 100) can be better indicators of sarcopenia [41–43]. However, after replacing the post-CRE in the top 4 features (*P* < 0.05) with the sarcopenia index and applying the same voting classifier, the classifier’s performance was not significantly improved. The voting classifier’s performance on two feature sets was shown in Table 6. It may be that CysC had no statistical difference in this study. Therefore, it did not help the model classify more accurately when it was combined with post-CRE.

**Table 6:** The voting classifier’s performance on two different top 4 feature sets for female MHD patients

Metrics	Top 4 features (*P* < 0.05)	Top 4 features (*P* < 0.05, post-CRE/CysC)
ACCTRS^a^	66.73% ± 4.28%	66.54% ± 3.87%
ACCTES^b^	74.29% ± 8.57%	71.43% ± 10.43%
AVAD^c^	12.72% ± 5.77%	11.38% ± 7.08%
Precision	81.86% ± 7.58%	79.35% ± 7.82%
Sensitivity	76.15% ± 13.95%	72.31% ± 13.85%
Specificity	71.25% ± 15.86%	70.00% ± 10.00%
F1 Score	78.04% ± 8.85%	75.25% ± 10.40%
AUC	77.69% ± 7.92%	78.37% ± 9.11%

^a^Accuracy of Training Set

^b^Accuracy of Test Set

^c^Absolute Value of Accuracy Difference between training set and test set

Meanwhile, previous studies have rarely discussed whether the pre-CRE or post-CRE is more useful. MHD patients are required to measure pre-CRE and post-CRE before and after dialysis, respectively. Hence, the use of a combination of these two indicators may bring new breakthroughs. The creatinine generation rate calculated from pre-dialysis and post-dialysis blood examinations positively correlates with skeletal muscle mass [44]. However, this study only used data directly extracted from the medical system and did not conduct related research due to limited time and effort.

In order to obtain a simple classification model, the number of features less than six was only considered. Other features may also be related to sarcopenia identification. For example, the relationship between dialysis duration and sarcopenia varies with study cohorts [7, 45]. Limited by the small data size, population, and requirements for model convenience, some features showed little or no help in identification. But they can be involved or replace some existing features in classifiers if appropriate.

### Limitation and development

The primary task of the classifier is to divide MHD patients into non-sarcopenia and sarcopenia groups. Generally, failing to identify a patient with sarcopenia leads to subsequent adverse events and prognoses, greatly destroying the doctor-patient relationship and making the patient question the classifier’s ability. Therefore, finding features with high sensitivity is the main aim of the best feature set selection. However, clinical diagnosis always pursues both higher sensitivity and specificity as much as possible. Hence, better feature sets and models still need to be found in further research.

Studies have shown that sarcopenia and frailty share many commonalities in the proposed underlying mechanisms involving a complex interplay between multiple systems and pathophysiologic processes [46]. Furthermore, physical performance is a common item to assess them. Sarcopenia and frailty are interrelated and both can increase the risk of adverse outcomes and mortality [6, 47]. Therefore, further understanding of the potential relationship between sarcopenia and frailty in MHD patients may help identify and diagnose these two diseases simultaneously. However, this study only focused on sarcopenia and considered two common comorbidities (DM and hypertension). Whether frailty could help identify sarcopenia was not considered. The role of frailty states in sarcopenia identification assistant tools for MHD patients remains unclear. Therefore, some convenient and available clinical frailty assessment results, such as clinical frailty scale results and hospital frailty risk score [48], should be included in data collection and model development in future research.

In this study, the voting classifier combing LR, Adaboost, and LGBM achieved an average AUC of more than 85% with only three features for males. However, the performance of SVM was the only acceptable one for females. The SD of the voting classifier’s metrics after stratified shuffle split were a little large because of the relatively small amount of data in one single hemodialysis center. Integrated tree-based models, which are more suitable for processing a large number of high-dimensional data [16], performed not as well as LR and SVM based on relatively simple theories. However, with the increase of the data amount, integrated tree-based models may show their advantages. Meanwhile, if there are big data available, deep learning models may be able to get better classification results.

Currently, there is no unified diagnostic standard for sarcopenia. Owing to the study population and geographical area limitations, this study only used AWGS 2019 as the criterion for sarcopenia diagnosis and true sample labels for model evaluation. This study aims to develop a sarcopenia identification tool for MHD patients, not a new diagnostic method. Therefore, the results may only be applied to populations who use the same consensus. It is still necessary to check the classifiers’ performance on other sarcopenia consensuses, and multi-regional or multi-center research.

## Conclusion

In this study, two simple sex-specific sarcopenia identification assistant tools for MHD patients using machine learning methods were developed. They performed well on the case finding of sarcopenia, especially possible sarcopenia assessed by the methods proposed in AWGS 2019. They can promote the early diagnosis and identification of sarcopenia among MHD patients and improve their quality of life. Limited by the single hemodialysis center research, the data size was relatively small after grouping by sex, and classifiers have not been applied in clinical practice. More samples are needed in further research. Moreover, the models and features that voting classifiers use should be adjusted to make classifiers more robust. The classifiers’ results on other sarcopenia consensuses, regions and populations should be verified, too.

## Data Availability

The datasets used and/or analysed during the current study are available from the corresponding author on reasonable request.

## Abbreviations

MHD: Maintenance hemodialysis
CKD: Chronic kidney disease
AWGS: Asia Working Group for Sarcopenia
SD: Standard deviation
IQR: Interquartile range
KNN: K-Nearest Neighbor
GaussianNB: Gaussian Naive Bayes
LR: Logistic Regression
SVM: Support Vector Machine
MLP: Multi-layer Perceptron
DT: Decision Tree
RF: Random Forest
Adaboost: Adaptive Boosting
GBDT: Gradient Boosting Decision Tree
LGBM: Light Gradient Boosting Machine
SMOTE: Synthetic Minority Oversampling Technique
TNR: True-negative rates
TPR: True-positive rates
AUC: The area under the receiver operating characteristic curves
DM: Diabetes mellitus
HTN: Hypertension
AVF: Arteriovenous fistula
CVC: Central venous catheter
DM: Dialysis duration
SARC-F: Strength,assistance with walking,rise from a chair,climb stairs and falls
CC: Calf circumference
WC: Waist circumference
HC: Hip circumference
AWVA: Arm without vascular access
HGS: Handgrip strength
SMI: Skeletal muscle index
BMI: Body mass index
RBC: Red blood cell count
HGB: Hemoglobin
HCT: Hematocrit
MCV: Mean corpuscular volume
MCH: Mean corpuscular hemoglobin
MCHC: Mean corpuscular hemoglobin concentration
RDW-CV: Red blood cell distribution width—coefficient of variation
RDW-SD: Red blood cell distribution width—standard deviation
PLT: Platelet count
WBC: White blood cell count
NEUT%: Neutrophil percentage
LY%: Lymphocyte percentage
MO%: Monocyte percentage
EOS%: Eosimophil percentage
BASO%: Basophil percentage
ANC: Absolute neutrophil count
ALC: Absolute lymphocyte count
AMC: Absolute monocytes count
AEC: Absolute eosinophils count
ABC: Absolute basophil count
TBIL: Total bilirubin
DBIL: Direct bilirubin
IBIL: Indirect bilitubin
ALT: Alanine amiotransferase
AST: Aspartate aminotransferase
AST/ALT: Aspartate aminotransferase/Alanine amiotransferase
TP: Total protein
ALB: Albumin
GLOB: Globulin
A/G: Albumin/globulin
FBG: Fasting blood glucose
pre-URE: Pre-dialysis urea
pre-CRE: Pre-dialysis creatinine
CysC: Cystatin C
UA: Uric acid
TG: Triglyceride
CHOL: Cholesterol
HDL-C: High density lipoprotein cholesterol
LDL: Low density lipoprotein
ALP: Alkaline phosphatase
GGT: γ-Glutamyl transpeptadase
s-Na: Serum sodium
s-K: Serum potassium
s-Cl: Serum chloride
CO_2_CP: Carbon dioxide combining power
AG: Anion gap
β-HBA: Serum β-hydroxybutyrate
s-Ca: Serum calcium
s-Mg: Serum magnesium
s-IP: Serum inorganic phosphorus
s-I: Serum iron
TIBC: Total iron binding capacity
TSAT: Transferrin saturation
PTH: Parathyroid hormone
s-Fe: Serum ferritin
CRP: C-reactive protein
β2-MG: β2-Microglobulin
post-URE: Post-dialysis urea
post-CRE: Post-dialysis creatinine
mCI: Modified creatinine index
