# Correction: Use machine learning to help identify possible sarcopenia cases in maintenance hemodialysis patients

**DOI:** 10.1186/s12882-023-03139-9

**Published:** 2023-04-26

**Authors:** Hualong Liao, Yujie Yang, Ying Zeng, Ying Qiu, Yang Chen, Linfang Zhu, Ping Fu, Fei Yan, Yu Chen, Huaihong Yuan

**Affiliations:** 1 Department of Nephrology, West China Hospital, Sichuan University/ West China School of Nursing, Sichuan University, Chengdu, 610041 Sichuan China; 2 Department of Applied Mechanics, College of Architecture and Environment, Sichuan University, Chengdu, 610065 Sichuan China; 3 Kidney Research Laboratory, Division of Nephrology, West China Hospital of Sichuan University, Chengdu, 610041 Sichuan China; 4 Chongqing Municipality Clinical Research Center for Geriatric Diseases, Chongqing University Three Gorges Hospital, School of Medicine, Chongqing University, Chongqing, 404000 China

**Correction: BMC Nephrol 24, 34 (2023)**

**https://doi.org/10.1186/s12882-023-03084-7**

Following publication of the original article [1], we have been informed that authors Hualong Liao, Yujie Yang, Ying Zeng, Ying Qiu, Yang Chen, Linfang Zhu, Yu Chen and Huaihong Yuan were incorrectly affiliated. Also, we have been informed that the spelling 'F1 Sore' from Fig. 3 is wrong.

The correct Fig. 3 is given below:

**Fig. 3:**
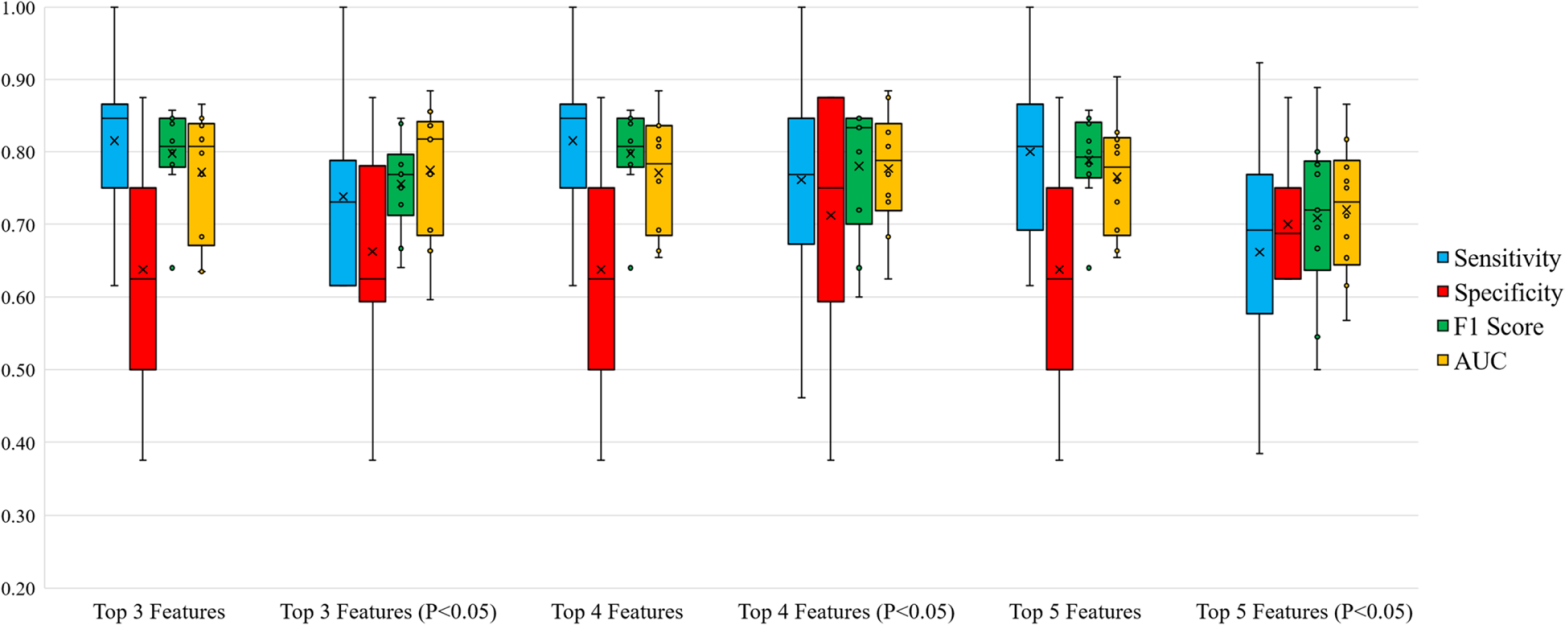
The box plot of voting classifier’s evaluation metrics about six feature sets in females. ×: the mean value mark. ○: the outliers

The authors affiliation has been updated above and the original article has been corrected.
